# Longitudinal study of humoral immunity to bovine coronavirus, virus shedding, and treatment for bovine respiratory disease in pre-weaned beef calves

**DOI:** 10.1186/s12917-019-1887-8

**Published:** 2019-05-22

**Authors:** Aspen M. Workman, Larry A. Kuehn, Tara G. McDaneld, Michael L. Clawson, John Dustin Loy

**Affiliations:** 1 United States Department of Agriculture (USDA) Agricultural Research Service (ARS), US Meat Animal Research Center (USMARC), State Spur 18D, Clay Center, NE 68933 USA; 2 Nebraska Veterinary Diagnostic Center, School of Veterinary Medicine and Biomedical Sciences, University of Nebraska-Lincoln, 4040 E Campus Loop, Lincoln, NE 68503 USA

**Keywords:** Bovine coronavirus, Bovine respiratory disease, *Histophilus somni*, Molecular epidemiology, Nursing-calf pneumonia, Summer pneumonia

## Abstract

**Background:**

Bovine coronavirus (BCV) is associated with respiratory infections in cattle of all ages; however, a temporal study to evaluate the effect of BCV immunity on virus shedding and bovine respiratory disease (BRD) incidence in pre-weaned beef calves has not been reported. Thus, we report here a prospective study in three herds of crossbred beef calves (*n* = 817) with endemic BCV.

Serial blood samples for measurement of serum anti-BCV antibody titers and nasal swabs for detection of BCV and other common viral and bacterial BRD pathogens were collected from all calves or subsets of calves at predetermined times from birth through weaning. The calves were monitored for BRD and those that developed signs of respiratory disease were sampled for diagnostic testing. To discover additional risk factors that could have influenced BRD development, sequence analysis of the BCV strain(s) circulating in each herd, and the prevalence of common opportunistic bacterial pathogens in the upper respiratory tract of sick and apparently healthy cattle were also evaluated.

**Results:**

Two hundred forty-eight of the 817 study calves (30.4%) were treated for BRD prior to weaning; 246 of those were from a single herd involved in two outbreaks of BRD leading to mass treatment of all calves in that group. Molecular diagnostic testing found BCV and *Histophilus somni* in nasal swabs taken at the time of BRD treatment. Between herd analyses revealed anti-BCV serum antibody abundance did not associate with the incidence of BRD or BCV shedding, though these measurements may have been hindered by the long periods between sample collections. Analysis of the BCV spike gene hypervariable region revealed four polymorphisms in 15 isolates from the three herds, making strain variation unlikely to account for differences in treatment rates between herds. Persistent or recurrent shedding episodes of BCV occurred in some animals treated for BRD.

**Conclusion:**

Co-detection of BCV and *H. somni* at the time of the disease outbreak suggests that these pathogens contributed to disease pathogenesis. Developing appropriate control measures for respiratory BCV infections may help decrease the incidence of pre-weaning BRD. The role of antibodies in protection must still be further defined.

**Electronic supplementary material:**

The online version of this article (10.1186/s12917-019-1887-8) contains supplementary material, which is available to authorized users.

## Background

Bovine respiratory disease (BRD) is the leading cause of morbidity and mortality for all production classes of cattle and calves in the U.S., causing losses to the cattle industry in excess of $1 billion dollars annually [1, 2]. Multiple etiologies, including both viral and bacterial, contribute to BRD [3]. Those generally accepted to be important contributors to BRD include the viral pathogens bovine herpesvirus-1 (BHV-1), bovine viral diarrhea virus types 1 and 2 (BVDV), bovine respiratory syncytial virus (BRSV) and parainfluenza-3 virus (PI3); and the bacteria *Mannheimia haemolytica, Pasteurella multocida, Histophilus somni* and *Mycoplasma bovis* [2, 4]. BRD is frequently initiated by a viral infection that disrupts local defenses and/or causes immune suppression, allowing opportunistic bacterial pathogens that are in healthy animals as normal nasophayngeal commensals to proliferate and infect the lungs [2, 4]. Superimposed environmental or management related stress (such as adverse weather, shipping, and commingling) can further suppress the host immune system, increase pathogen exposure, and may be important co-requisites in many BRD outbreaks [4]. Although vaccines and antibiotic treatments are readily available to prevent and treat infection caused by common BRD pathogens, the incidence of disease remains high [5].

In recent years, bovine coronavirus (BCV) has been implicated as an important contributor to BRD [6]. Although initially described as being associated with calf diarrhea, BCV has been found to infect the upper and lower respiratory tract and has been isolated from pneumonic lungs alone or in combination with other respiratory pathogens [7–12]. In addition, results of multiple studies indicate that groups of cattle with high titers of serum antibodies to BCV at the time of feedlot entry are less likely to shed BCV and develop BRD than those with low anti-BCV serum antibody titers [7, 13–15]. Taken together, it appears that BCV contributes to feedlot BRD, and high titers of serum anti-BCV antibodies associate with reduced risk of BCV infection and disease. However, it remains unknown whether the serum antibodies themselves are immune correlates of protection, or whether they simply reflect prior exposure to the virus [6].

The relationship between BCV and BRD in pre-weaned beef calves has not been comprehensively evaluated. Though BCV is frequently detected in nasal swabs from nursing calves with BRD, subclinical BCV infections are also common in young dairy calves, even in the presence of relatively high anti-BCV antibody titers [16, 17]. These results raise questions about the association between anti-BCV antibody titers and BCV shedding with the risk of developing BRD in nursing dairy calves. Similarly, in a 2014 study, our group sampled four research herds (*n* = 890) at predefined times from birth through their fifth week in the feedlot [15]. This study revealed that the herds in which BCV was detected in nasal sections during the pre-weaning period also had the highest incidence of pre-weaning BRD; however, nasal swabs were not collected at the time of treatment to diagnose the pathogens associated with those pre-weaning BRD cases. This study also reported that serum anti-BCV antibody abundance did not correlate with BCV shedding prior to weaning. Thus, while mounting evidence suggests that anti-BCV antibodies protect weaned feedlot cattle from BRD associated with BCV infection, the relationship between humoral immunity to BCV, virus shedding, and the risk for developing BRD in nursing calves remains unclear. This represents a major obstacle in the development of effective control strategies to reduce the impact of BCV-related respiratory disease in cattle, which is significant given that there are currently no licensed BCV vaccines in the United States to aid in the prevention of BRD.

To address this knowledge gap, the present study serially sampled 817 calves from three herds of beef cattle from birth through weaning to determine whether shedding of BCV is associated with BRD and whether levels of anti-BCV serum antibodies associate with BCV shedding or BRD incidence in pre-weaned beef calves. Sequence analysis of the virus strain(s) circulating in each herd and the prevalence of common opportunistic bacterial pathogens (*M. haemolytica, P. multocida, H. somni* and *Mycoplasma bovis*) in the upper respiratory tract of sick and apparently healthy cattle were also evaluated to account for potentially confounding factors that could influence BRD development in these populations.

## Methods

### Ethical statement

All experimental procedures were performed with approval and under the guidelines of the US Meat Animal Research Center (USMARC) Institutional Animal Care and Use Committee (IACUC approval numbers 5438–31,000–082-04 (24) and 3040–32,000–031-07 (5)).

### Study population

**Fig. 1:**
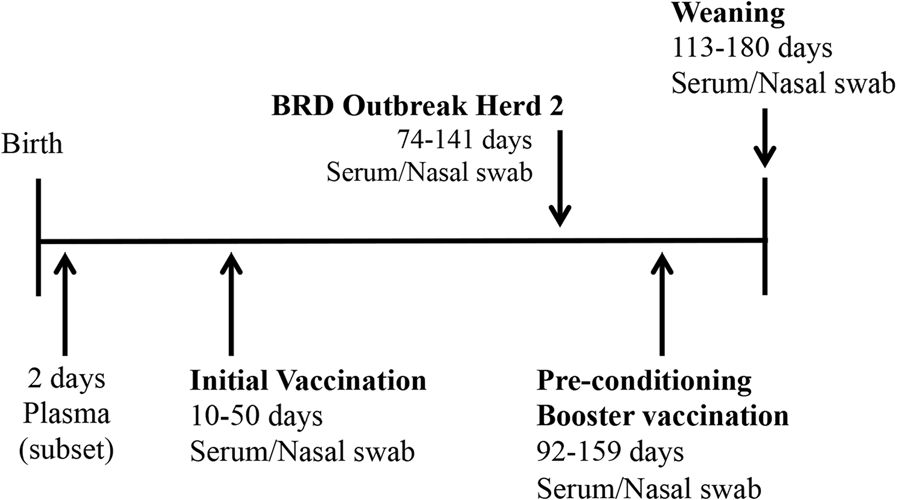
Timeline of calf vaccinations and sample collection. For vaccine details see Methods section. Of note, at no time during the study were calves vaccinated with a BCV containing vaccine. Mass treatment for BRD occurred in a subset of claves from Herd 2 on August 5, 2016 (mass treatment 1, MT-1; n = 93 study calves) and August 12, 2016 (mass treatment 2, MT-2; n = 142 study calves). Additional details are in the Methods

Eight hundred seventeen natural-service, crossbred beef calves born between March 24 and May 24, 2016, were followed from birth through weaning (Fig. 1). The calves were part of the ongoing germplasm evaluation program at the USMARC, located on approximately 35,000 acres near Clay Center, Nebraska, USA [18]. Calves were a product of multiple-sire matings of crossbred cows to F1 bulls of varying breed composition. The resulting calves used within this study consisted of variable fractions of 18 breeds: Angus, Hereford, Red Angus, Brahman, Charolais, Gelbvieh, Limousin, Simmental, Brangus, Beefmaster, Shorthorn, Maine Anjou, Santa Gertrudis, Chiangus, Salers, Braunvieh, South Devon, and Tarentaise.

Study calves originated from three research herds that were managed in separate locations at USMARC. The three herds did not have fence-line contact, so there was no direct pathogen transmission between herds. These calves were raised with similar management and received standardized vaccinations (as described below). Herd characteristics, including the number of calves enrolled in this study from each herd, median dam parity within each herd, and mean ± standard deviation weaning age and weight for the study calves were summarized in Table 1. Each of these three herds contained calves in addition to the target population enrolled in the study. Herds 1 and 2 consisted of calves that were born as a result of artificial insemination or natural service, whereas Herd 3 consisted of only calves that were born as a result of full-season natural service breeding. Only natural service calves from each herd were enrolled in the study because calves that were born as a result of artificial insemination were enrolled in another study on center. As result, there are differences in the mean age and weight of study calves at weaning (Table 1). The additional calves in each herd were managed identically to the target population but were not sampled for this study.

**Table 1:** Study population. Descriptive characteristics for 817 natural-service, crossbred beef calves born in three USMARC herds during the months of March through May, 2016

Variable	Herd		
	1	2	3
Total No. of calves	676	524	194
No. of calves enrolled in study	261	388	167
Median (range) dam parity	2.6 (2–4)	6.0 (3–9)	7.9 (6–11)
Average weaning age ± SD (days)	136 ± 13^a^	151 ± 14^b^	147 ± 14^c^
Average weaning weight ± SD (kg)	180 ± 22^a^	206 ± 28^b^	197 ± 25^c^

^a, b, c^Within a row, values with different superscripts differ significantly (*P* < 0.05)

### Vaccination schedule and calf processing

Dams: The dams in all three herds received a modified live virus (MLV) vaccine-bacterin combination product (Bovi-Shield Gold FP 5 VL 5, Zoetis, Florham Park, NJ) that contained MLV strains of BHV-1, BVDV types 1 and 2, PI3, BRSV, and an inactivated suspension of *Campylobacter fetus* and 5 strains of *Leptospira.* This vaccine was given annually, prior to breeding (approximately June, one to three weeks before breeding). The dams were also vaccinated against enteric pathogens approximately 30 days before the beginning of calving season. That vaccine (Scourguard 4KC; Zoetis) contained killed strains of bovine rotavirus and BCV, a K99 *Escherichia coli* bacterin, and *Clostridium perfringens* type C toxoid. This vaccine enhances the production and secretion of antibodies against those pathogens into the colostrum to aid in the prevention of diarrhea in calves. This is the only BCV containing vaccine used in the study.

Calves: A timeline of calf vaccinations is provided in Fig. 1. At approximately 10 to 50 days of age (initial vaccination), calves received a MLV vaccine-bacterin combination product (Bovi-Shield Gold One Shot, Zoetis) that contained MLV strains of BHV-1, BVDV types 1 and 2, PI3, BRSV, and *Mannheimia haemolytica* and a multivalent bacterin-toxoid product (Vision 7 with Spur, Merck, Kenilworth, NJ) intended to protect animals against disease caused by *Clostridium sp.* and *Moraxella bovis*. At preconditioning processing, (21 days prior to weaning; calves 92 to 159 days old), calves received a booster of the respiratory vaccine (Bovi-Shield Gold One Shot, Zoetis) and another multivalent clostridial vaccine (Vision 8, Merck). Calves were weaned into the feedlot at 113 to 180 days of age. Of note, at no time in the study did the calves receive a BCV containing vaccine.

### Sampling procedures

A timeline of sample collection is shown in Fig. 1 and the number and types of samples taken at each time point is summarized in Table 2. A 9 mL blood sample for plasma acquisition was collected between 18 and 48 h after birth from a small subset of calves for which both birth and the first nursing episode were observed (*n* = 169, [herd 1 = 33; herd 2 = 82; herd 3 = 54]). A 9–25 mL blood sample (for serum acquisition) and nasal swab specimens (for pathogen detection) were collected from all study calves (*n* = 817) at initial vaccination, preconditioning, and weaning. These three sampling periods were selected to minimize stress on the calves from excess handling, as calves were routinely processed and vaccinated at those production stages at USMARC. An additional 9 mL blood sample and nasal swab samples were collected from calves at the time of treatment for BRD during the study period (*n* = 234 calves sampled of 248 treatments).

**Table 2:** Summary of sample collection and testing performed

Collection time	Samples collected	Number sampled	Testing
Birth	Blood	Subset^a^, *n* = 169	*n* = 169 Maternal anti-BCV IgG
Initial vaccination	Blood, NS^b^	All study calves^c^	*n* = 60 each herd, 180 total Blood: anti-BCV IgG NS: subclinical virus shedding
Preconditioning	Blood, NS	All study calves	*n* = 60 each herd, 180 total Blood: anti-BCV IgG NS: subclinical virus shedding
Weaning	Blood, NS	All study calves	*n* = 60 each herd, 180 total Blood: anti-BCV IgG NS: subclinical virus shedding
At time of treatment for BRD	Blood, NS	*n* = 234 (including mass treatments)	Blood: anti-BCV IgG NS: detection of viral and bacterial pathogens

^a^Blood was collected between 18 and 48 h after birth from a small subset of calves for which both birth and the first nursing episode were observed to ensure chance for colostrum uptake

^b^NS, nasal swabs

^c^817 calves were sampled for this study, see Table 1

Blood samples were taken via jugular venipuncture using 18-gauge needles at birth and initial vaccination and 16-gauge needles at later sampling points. Plasma or serum were separated from other blood components via centrifugation (1650 x g, 25 min, 4 °C) and stored at − 80 °C. Nasal samples were collected from the upper nasal cavity of calves using sterile 6-in. cotton-tipped swabs. For sampling, the nasal swab was gently inserted into the nasal cavity at an approximate depth of 6 in., rotated, and removed. Nasal swabs were placed in cryovials containing 1 mL transport medium (buffered peptone water with 12% glycerol), snap frozen in liquid nitrogen, and stored at − 80 °C.

### Mass treatment in response to pre-weaning BRD outbreak

Two subsets of calves in Herd 2 (*n* = 235) were mass treated for BRD in August 2016, according to the USMARC standard operating procedure (SOP) for intervention in disease outbreaks (Fig. 1). At dam prebreeding processing (approximately June), the dams and their calves were split into three breeding groups based on dam age. During breeding, groups were separated by at least one pasture, so no breeding groups shared fence-line contact at this time. On August 5, 2016, calves in one breeding group (*n* = 93 study calves) were mass treated for BRD following the observation by the attending veterinarian that approximately 15–20% of the calves were displaying clinical signs of BRD, including: cough, nasal discharge, increased respiratory rate, lethargy, and anorexia. Calves were individually restrained in a squeeze chute for sample collection (as described above) and treatment (Draxxin (tulathromycin; macrolide), Zoetis) then returned to their pasture. On August 12, 2016, a second breeding group (*n* = 142 study calves) was mass treated with a different antibiotic (Zuprevo (tildipirosin; macrolide), Merck) following the observation that 25–30% of the calves in that pasture were displaying clinical signs of BRD. They were similarly sampled, treated, and returned to their pasture. All treatment decisions were made by the attending veterinarians and carried out according to SOP.

### Detection of viral pathogens by RT-qPCR

A multiplex reverse transcription real-time polymerase chain reaction (RT-qPCR) was used to detect BCV, BRSV, BVDV, and BHV-1 in nasal swab samples as previously described [15] using primer and probe sets from previously published reports [19–22]. Briefly, RNA was extracted from 250 μl of sample using a phenol and guanidine isothiocyanate reagent (TRIzol LS, Life Technologies, Grand Island, NY) and DNA was extracted from 200 μl of sample using a silica-membrane-based nucleic acid purification kit (QIAamp DNA mini kit, Qiagen Inc., Valencia, CA). Cyclic amplification reactions were carried out in a 25 μl reaction containing: 4.5 mM MgCl_2,_ 400 μM concentration of each dNTP, 0.4 μM concentration of each primer, 0.2 μM concentration of each probe, 1 μl enzyme mix containing reverse transcriptase (RT) and a hot start Taq polymerase (OneStep RT-PCR kit, Qiagen Inc.), and 2.5 μl each of RNA and DNA. Cycling conditions were as follows: reverse transcription for 30 min at 50 °C, inactivation of RT enzyme and activation of Taq polymerase for 15 min at 95 °C followed by 40 cycles of 94 °C for 30 s, 55 °C for 60 s and 72 °C for 60 s. Ct values < 40 were considered positive. Positive, negative, no template, and extraction controls were included on each run.

For swabs taken from Herd 2 calves at the time of mass treatment for BRD, 100 μL of transport medium from nasal swab samples were pooled together into groups of approximately five based on collection date and the calf’s rectal temperature at the time of treatment. This pooling strategy was utilized to determine whether there were differences in the prevalence of pathogens between calves with a normal rectal temperature and those with an elevated rectal temperature at the time of treatment for BRD. RT-qPCR results are presented for the pooled samples.

Subclinical shedding of viral pathogens was also assessed by RT-qPCR methods in 60 randomly selected study calves from each of the three herds at initial vaccination, preconditioning, and weaning. This sample size yielded a 95% probability of detecting at least one infected animal if the true shedding prevalence was at least 5% in the herd at that production stage [23]. At each sample acquisition time, 100 μL of transport medium from nasal swab samples were pooled together into groups of five based on originating herd. Samples from pools found to be positive for viral RNA or DNA were then processed individually to identify the positive individual(s) in each pool. RT-qPCR results are reported for the number of individuals identified as positive after testing all individuals in the positive pools.

### Detection of bacteria by qPCR

DNA extracted from pooled nasal swab medium was sent to the University of Nebraska-Lincoln Veterinary Diagnostic Center for bacterial diagnostics by multiplex qPCR using the QuantiFast multiplex PCR kit (Qiagen Inc.) and primers and probes designed to detect *M. haemolytica, P. multocida, H. somni* and *Mycoplasma bovis*. This test has been validated for use with bovine nasal swabs and lung tissue matrices [24].

### BCV antibody detection ELISA

Passively acquired immunity to BCV (maternal antibodies) was evaluated in a subset of calves from each herd (*n* = 169, [herd 1 = 33; herd 2 = 82; herd 3 = 54]) by measuring the abundance of serum anti-BCV IgG antibodies in the calf at 18–48 h after birth. BCV antibodies were also measured for each of the 180 calves (60 from each herd) that were evaluated for BCV shedding at initial vaccination, preconditioning, and weaning. Additionally, antibody levels were measured over time, from birth through weaning, in 39 calves from Herd 2 that were involved in the mass treatment event for pre-weaning BRD on August 12, 2016.

Antibody levels were measured using a commercially available indirect antibody ELISA (BCV antibody ELISA, Boehringer Ingelheim Svanova, Uppsala, Sweden) according to the manufacturer’s instructions, as previously described [15]. Briefly, optical densities (OD) were used to calculate the BCV antibody percent positivity, by dividing the OD of the unknown clinical sample by the OD of the positive control provided in the kit. As per the manufacturer’s specification, a percent positivity value of < 10 was considered to be seronegative, while > 10 was considered to be seropositive. However, because the goal of this study was to compare antibody abundance between animals and herds and correlate these values with virus shedding and disease incidence, we first experimentally determined the linear range of the kit [15]. A good linear range from OD 0.1–1.5 was demonstrated. When measured values were compared to expected values, the linearity was found to fit the following equation: Y = 1.657(X)-0.08916, R^2^ = 0.999. All samples that were above an OD 1.4 were diluted further and retested. The adjusted percent positivity was then calculated based on the dilution factor of the clinical sample times the percent positivity. This value was accepted as the relative anti-BCV antibody abundance. The kit sensitivity is estimated to be 84.6% and the specificity is reported to be 100% [25, 26]. To reduce variation, kits of the same lot number were used for all tests.

### BCV neutralizing antibody detection by a virus neutralization test

A microtitration virus neutralization test (VNT) was used to determine titers of neutralizing anti-BCV antibodies in a subset of serum and plasma samples (*n* = 60). The goal was to evaluate the relationship between total anti-BCV reactive antibodies measured by ELISA and neutralizing antibody titers measured by a VNT. For these tests, HRT-18G cells (ATCC CRL11663) were grown and maintained in MEM (Gibco, Thermo Fisher Scientific, Waltham, MA) supplemented with 10% fetal bovine serum (Atlas Biologicals, Fort Collins, CO), 1x antibiotic-antimycotic (Gibco), and 2 mM L-glutamine (Gibco). Cells were seeded in 96-well plates at 8 × 10^3^ cells/well and incubated at 37 °C for 4 days or until cells were just confluent. Cells were then washed two times with diluent #5 (MEM supplemented with 1x antibiotic-antimycotic and 1% NaHCO_3_ [27];) and then incubated at 37 °C for 3 h in 100 uL diluent #5. During this incubation, serial 2-fold dilutions of the serum/plasma samples were made in MEM in a separate 96-well plate using duplicate rows for each sample. The challenge virus (described below) was diluted in MEM to contain 100 tissue culture infective dose_50_ per well (100 TCID_50_/well) and was added to the serum. The virus and serum were incubated for one hour at 37 °C before being transferred to the HRT-18G cells. Plates were incubated for 4–5 d in the 37 °C incubator. The endpoint titer was the final serum/plasma dilution that completely inhibited the viral cytopathic effects in both wells. Sera were tested in duplicate with known negative control sera in the assay.

Two challenge strains were used for the VNT: the cell culture adapted US reference enteric strain, Mebus (kindly provided by Dr. Linda Saif; The Ohio State University, Columbus, OH); and a respiratory strain isolated in 2014 from a naturally-infected calf in the same research herd as the serum samples to be tested (BRCV_2014). BRCV_2014 was propagated six times in HRT-18G cells. The Mebus virus strain has been passaged in multiple cell types and the passage number was unknown.

### BCV spike gene sequencing

A total of 15 BCV-positive nasal swab samples were sequenced directly from non-cultured clinical samples: six were from Herd 2 at the time of mass treatment, eight from preconditioning (two from Herd 1, four from Herd 2 and two from Herd 3), and one from weaning (Herd 2). Prior to RNA extraction, nasal swabs were treated with RNase and DNase to deplete host and contaminating environmental nucleic acid as previously described [28, 29]. The remaining nucleic acid was extracted using Trizol LS (Life Technologies). A nested PCR was then used to amplify a 1102 bp fragment of the spike gene, including the hypervariable S1 region used for phylogenetic analysis of coronavirus stains [30]. The primers were: BCV Forward: 5′-GATAAGTTTGCAATA-CCCAATGG-3’ [31], BCV Reverse: 5′-GTAAACCGATAACCAGTGG-3’ [30], Nest Forward: 5′-TGCAATACCCAATGGTAGG-3′, Nest Reverse: 5′-TGTAGAGTAATCCACACAGT-3′ [32]. A 5 μl sample of the final PCR product was run on a 1% agarose gel to confirm amplification of the 1102 bp product. These fragments were then purified from the remaining PCR sample by exonuclease treatment (Exo I, Life Technologies, Grand Island, NY) according to the manufacturer’s specifications. DNA was then precipitated with ethanol and sequenced twice in the forward direction and twice in the reverse direction using the above nested coronavirus primers at a commercial sequencing facility (Genewiz, South Plainfield, NJ). Resulting sequence data was then analyzed and edited using commercial software (Geneious, version 9.1.8, Biomatters, Auckland, New Zealand). Sequence alignments were carried out using MUSCLE [33] within Geneious. Single nucleotide polymorphism (SNP) locations were determined manually by comparison with the BCV strain Mebus reference genome (accession number U00735.2).

### Statistical analysis

#### Analysis of variance (class regression) models for differences in weight, age, and maternal antibodies

Differences between weaning weights, weaning ages, or maternal BCV IgG antibodies (log) across herds were analyzed in a model where location was fixed and animals were the residual.

#### Analysis of variance (class regression) models for differences in bacterial prevalence

Bacterial prevalence (yes or no) in the outbreaks and bacterial abundances were tested relative to body temperature above or below 39.4 °C (103 °F) in a model that included the mass treatment event (MT-1 or − 2), temperature status (above/below 39.4 °C) and their interaction. Due to the binary nature of prevalence as a response variable, a logistic generalized model was fitted to prevalence.

#### Analysis of variance (class regression) models for differences in BCV IgG serum antibody levels (log)

Changes in BCV IgG serum antibody levels (log) over time were modeled as repeated measures with unstructured multinomial covariances between the residuals at different time points. Location, time, and their interaction were tested as fixed in this model. Within herd two, where the BRD outbreaks occurred, breeding group was fitted as fixed rather than location to track differences in the groups that were mass treated. Finally, to examine the relationship between BCV shedding and anti-BCV IgG antibody levels in different herds, IgG level (log) was modeled within timepoints with fixed effects of location, shedding status, and their interaction and animal as the residual.

#### Quantification of the relationship between VNT and ELISA

Pearson’s correlation coefficients were derived to describe the linear relationship between the results obtained by VNT and ELISA and between the VNT using Mebus and BRCV 2014 challenge strains.

## Results

### BRD treatment

Two hundred forty-eight of the 817 study claves (30.4%) were treated for BRD prior to weaning. Two of these calves were from Herd 1 and the remaining 246 cases were from Herd 2. No study calves from Herd 3 were treated for BRD. No co-morbidity such as diarrhea was observed in BRD cases, and no study calves were treated for diarrhea at any point during the study.

The number of calves treated for BRD is inflated for Herd 2 because two subsets of calves from this herd (sorted into breeding groups at this time based on dam age) were involved in outbreaks of BRD leading to mass treatment of all calves in that group (*n* = 235; Methods and Fig. 1). At the time of the first mass treatment (MT-1), 22 of the 93 study calves (24%) had a rectal temperature ≥ 39.4 °C (103 °F), which was a threshold indication that an infectious or inflammatory process was occurring in the calves. The mean age of the calves at the time of MT-1 was 94 days (range: 74–126 days). The calves involved in the second mass treatment (MT-2) had a mean age of 113 days (range: 81–141 days) and 44 of the 142 calves (44%) had a rectal temperature ≥ 39.4 °C at the time of treatment. The remaining 11 cases from Herd 2 were treated outside the time of the mass treatment events and samples/rectal temperatures were not obtained. Five of those cases were from the third breeding group that was not mass treated for BRD (Additional file 1).

### Pre-weaning BRD cases were associated with the detection of BCV in nasal swab specimens

**Fig. 2:**
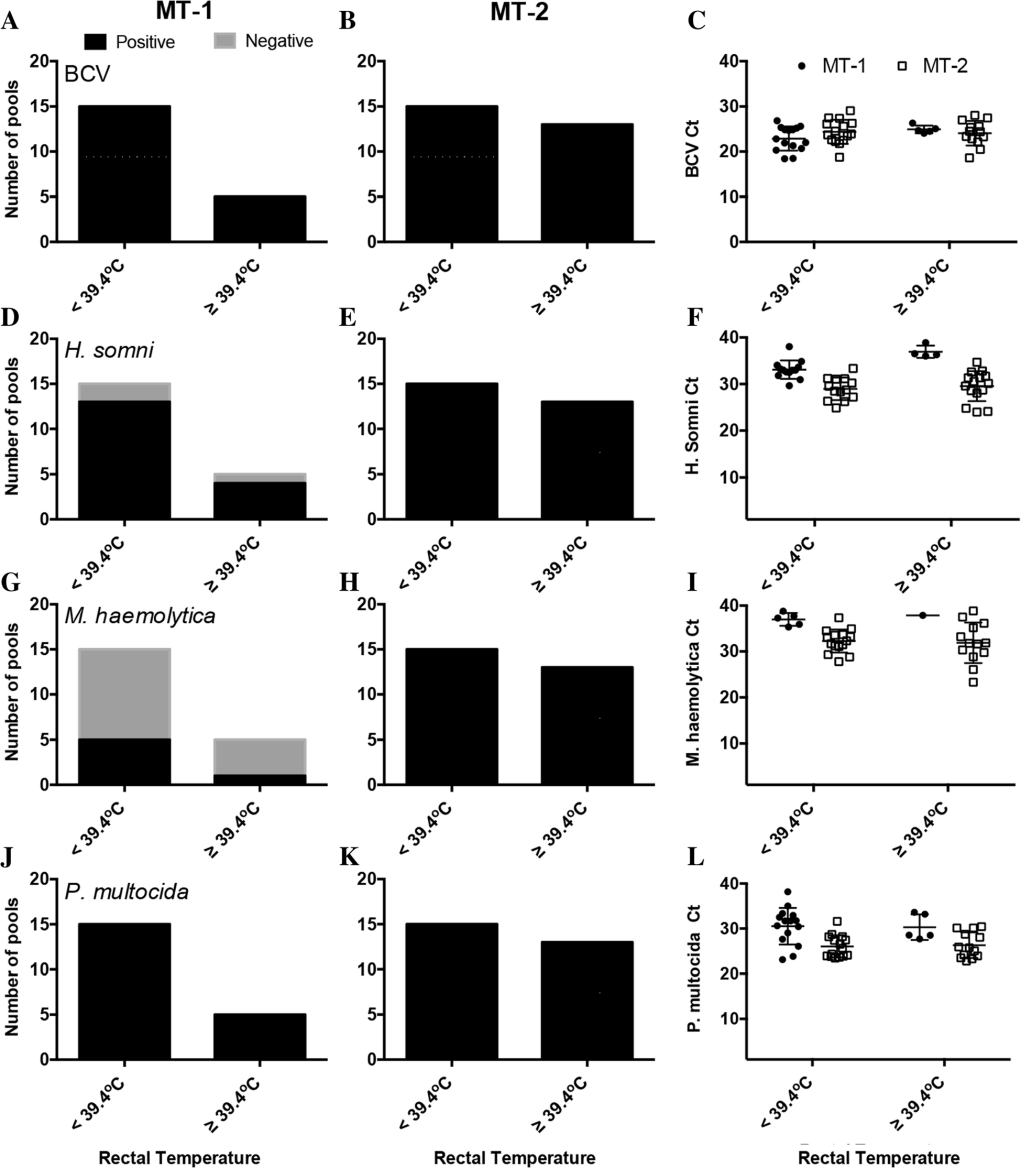
Prevalence and relative abundance of respiratory pathogens at the time of treatment for BRD. The prevalence and relative abundance of respiratory pathogens in the upper respiratory tract of calves from Herd 2 was determined by RT-qPCR (BCV) or qPCR (bacterial pathogens) in 48 pools of nasal swabs from 234 calves treated for BRD. Numbers of positive and negative pools for each respiratory pathogen detected in nasal swab specimens collected August 5 (MT-1) are shown in the first column (panels **a**, **d**, **g**, and **j**; 15 pools < 39.4 °C and 5 pools ≥39.4 °C) and those collected on August 12 (MT-2) are shown in the second column (panels **b**, **e**, **h**, and **k**; 15 pools < 39.4 °C and 13 pools ≥39.4 °C). The third column (panels **c**, **f**, **i**, and **l**) shows the cycle threshold (Ct) values for PCR positive pools. The Ct value is inversely associated with the amount of pathogen RNA or DNA detected; therefore, the lower the Ct value, the greater the amount of pathogen specific nucleic acid was present in the nasal swab specimen. A Ct < 40 was considered positive for all pathogens

Molecular diagnostics were carried out on 234 nasal swab specimens collected from Herd 2 at the time of mass treatment to detect some of the most common viral (BCV, BHV-1, BVDV and BRSV) and bacterial (*M. haemolytica, P. multocida, H. somni* and *M. bovis*) pathogens associated with BRD. BCV was detected in 48 of 48 pools of nasal swabs across both treatment dates. No statistically significant difference in relative abundance (as determined by RT-qPCR cycle threshold; Ct) was detected between treatment dates or between those with a rectal temperature ≥ 39.4 °C and those with normal rectal temperature within treatment dates (Fig. 2a-c). No other respiratory viral pathogens were detected.

Opportunistic bacterial pathogens *H. somni, M. haemolytica,* and *P. multocida* were detected in nasal swabs from calves across both treatment dates as summarized in Fig. 2. There were differences in both the prevalence and relative abundance of *H. somni* and *M. haemolytica* between the two treatment dates, and a difference in *P. multocida* abundance (*P* < 0.05). Within treatment dates, there was no statistical difference in the relative abundance of these bacteria when analyzed by rectal temperature. *M. bovis* was not detected in any nasal swab specimen.

**Fig. 3:**
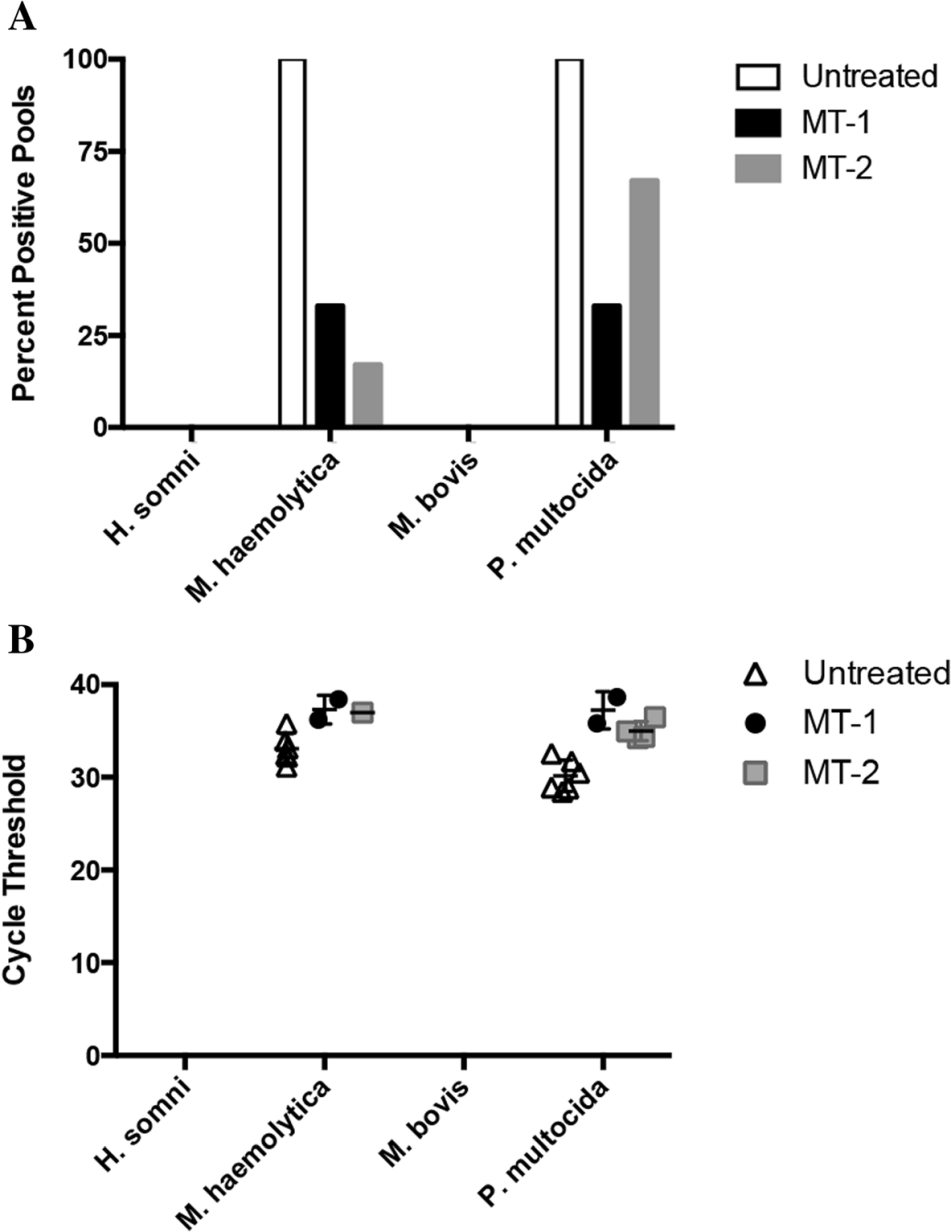
Prevalence and relative abundance of respiratory pathogens in the upper respiratory tract at preconditioning processing. Within each group, six pools of nasal swabs, with five animals per pool, were tested for opportunistic bacterial pathogens at preconditioning processing by qPCR for groups that were treated previously for BRD (MT-1 and MT-2) or those that remained untreated. **a** Number of pools positive for each bacterial species at preconditioning processing. **b** Cycle threshold for qPCR positive pools

Given that these bacteria are commensals found in the upper respiratory tract (URT) of clinically normal animals, and samples were not collected from apparently healthy controls outside of the treatment groups, interpretation of these results can be challenging. Therefore, the presence of these bacteria was determined two weeks (MT-2) or three weeks (MT-1) after the outbreak at routine preconditioning processing in untreated and previously treated calves from the same herd to help determine which (if any) bacteria may have been associated with the BRD outbreak (Fig. 3). Interestingly, at the time of mass treatment, *H. somni* was detected in 94% of the pools tested across both treatment dates (Fig. 2). At preconditioning, there was no evidence of *H. somni* in the recovered calves previously treated for BRD or in control calves from the same herd that were not involved in the mass treatment events (Fig. 3a). Furthermore, there was no evidence of *H. somni* in the nasal cavity of these same calves at initial vaccination, which was prior to the BRD outbreak (data not shown).

*M. haemolytica* and *P. multocida* were also detected at high levels in the calves treated for BRD; however, these bacteria were also detected in 100% of the pools at preconditioning from clinically normal calves not previously treated for BRD (Fig. 3a). There was, however, a statistically significant increase (*P* = 0.0011) in the abundance of *P. multocida* in the upper respiratory tract of cattle at the time of MT-2 compared to the untreated group at preconditioning processing (data not shown). Unsurprisingly, systemic antibiotic therapy in calves likely altered the bacterial profile in the upper respiratory tract of treated animals, as evidenced by the reduced prevalence (Fig. 3a) and abundance (Fig. 3b) of these bacteria in the URT at preconditioning compared to untreated controls.

### Anti-BCV serum antibody abundance measured over time in cattle treated for BRD

**Fig. 4:**
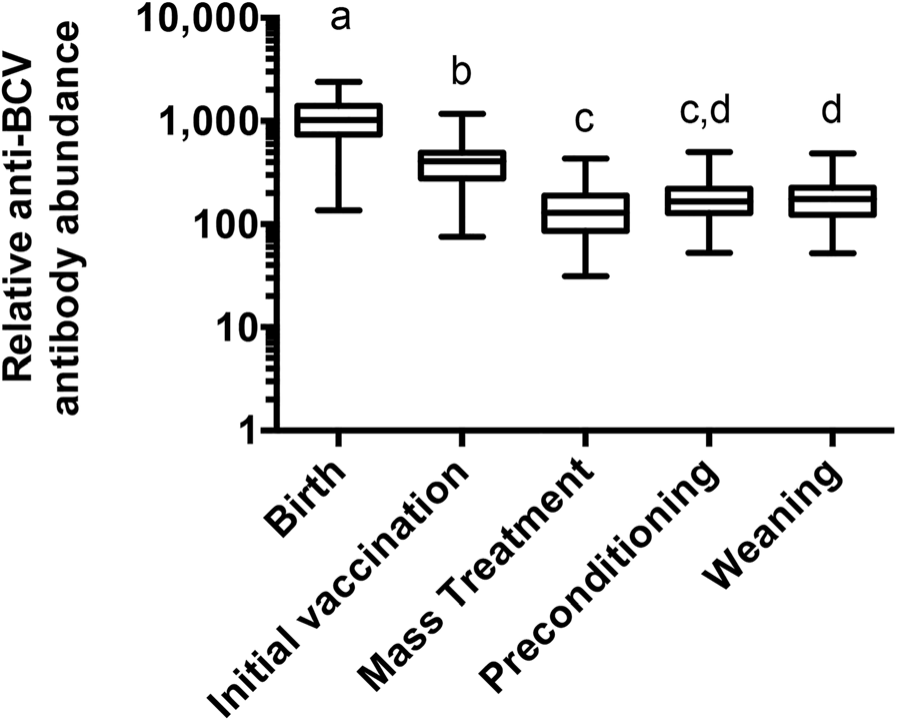
Anti-BCV antibody levels measured over time in calves treated for BRD. Anti-BCV antibody levels were measured by ELISA in 39 calves from Herd 2 that were involved in mass treatment for BRD on August 12, 2016 (MT-2). Differences in mean antibody abundance were determined based on contrasts of least-squares means being different from zero in models with sampling time as a fixed effect. Sample collection times with the same superscript are not significantly different (*P* > 0.05) in these contrasts

Serum anti-BCV IgG antibodies were measured by ELISA in 195 samples collected from birth through weaning for 39 calves from Herd 2 that were involved in the mass treatment for BRD on August 12, 2016 in order to determine the mean antibody abundance and range at each sample acquisition time (Fig. 4). The mean (± standard deviation) anti-BCV antibody abundance declined from a maximum of 1186 ± 699 at birth to a low of 138 ± 88 at the time of mass treatment. Mean antibody abundance increased slightly following mass treatment, with mean antibody abundances of 176 ± 83 and 182 ± 95 at preconditioning and weaning, respectively.

Neutralizing antibody titers were measured in 60 of these samples from 12 randomly selected calves to determine the relationship between total anti-BCV reactive antibodies measured by ELISA and neutralizing antibody titers measured by a virus neutralization test (VNT). The effect of altering the strain of the test virus used in the VNT was also evaluated. A high positive mean correlation was observed between the ELISA and VNT assays regardless of the test virus used (Pearson’s rank correlation, ρ = 0.81 with BRCV_2014 strain and ρ = 0.91 with Mebus strain), indicating good to excellent agreement between the two tests under these conditions (Additional file 2). Thus, the ELISA was used for subsequent measurements of anti-BCV antibodies.

### Anti-BCV serum antibody abundance did not associate with pre-weaning BRD incidence

**Fig. 5:**
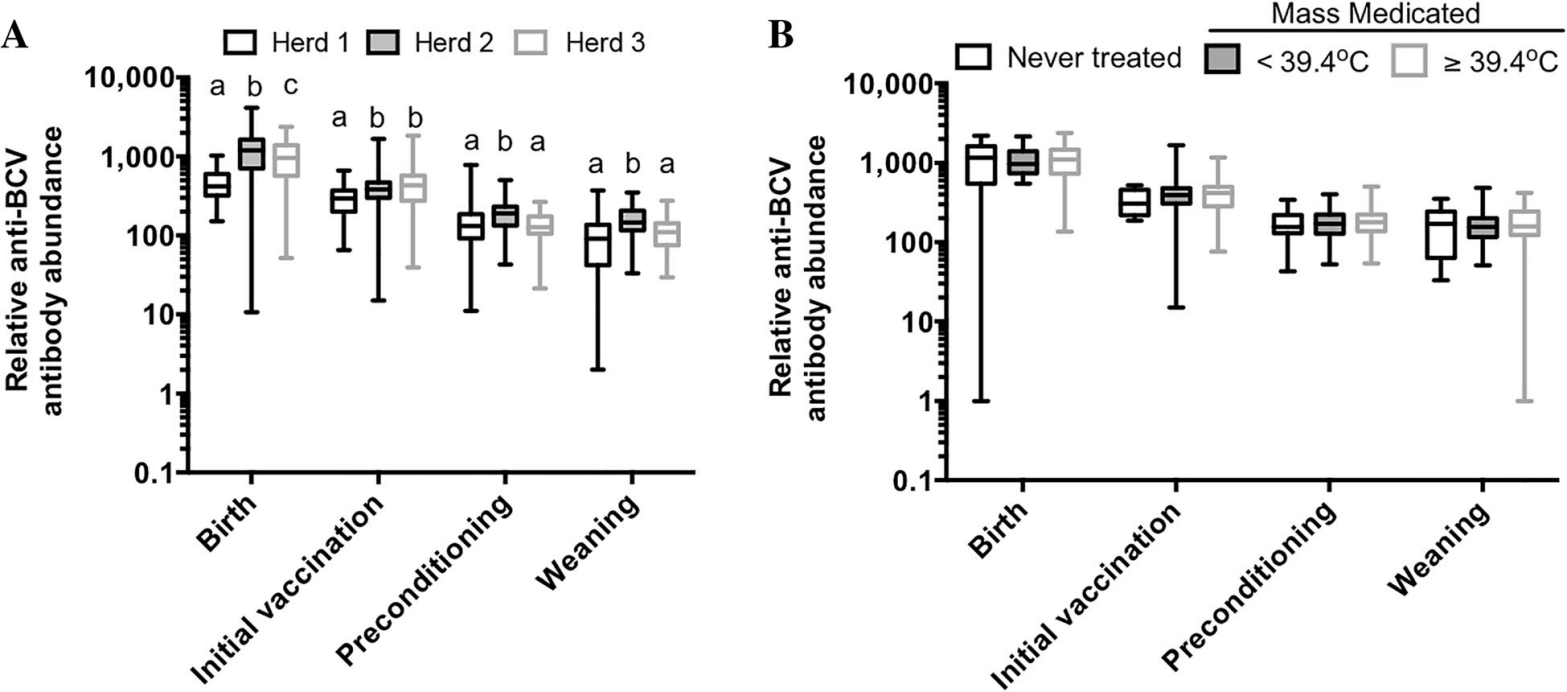
Relationship between anti-BCV antibody abundance and BRD. **a** Passively acquired immunity to BCV (maternal antibodies) was evaluated in a subset of calves from each herd (*n* = 169, [herd 1 = 33; herd 2 = 82; herd 3 = 54]) by measuring the abundance of serum anti-BCV antibodies in the calf at 18–48 h after birth. At initial vaccination, preconditioning and weaning, anti-BCV antibody levels were determined in serum samples from 60 randomly selected calves from each herd to determine the mean and range of antibodies in each herd at each production stage. **b** Anti-BCV antibody levels for calves from Herd 2 (from Figs. 4 and 5a) were re-plotted based on rectal temperature at the time of mass treatment for BRD: those mass treated for BRD with a rectal temperature ≥ 39.4 °C (*n* = 14 at birth and 27 all other time points) and those mass treated for BRD with a rectal temperature < 39.4 °C (*n* = 25 at birth and 49 at all other time points). These values were compared to those from animals of the same herd that remained untreated for BRD (*n* = 42 at birth and 15 at all other time points). Differences in mean antibody abundance were based on contrasts of least-squares means being different from zero in models with location and time as fixed effects (repeated measures); contrasts were evaluated if the overall *p* value for location was significant. Herds with the same superscript were not significantly different (*P* > 0.05) in these contrasts

To determine whether levels of passively acquired (maternal) antibodies were associated with pre-weaning BRD incidence, relative anti-BCV antibody abundances were measured in a subset of calves from each herd at 18–48 h after birth. Herds 1–3 had mean ± SD antibody abundances of 473 ± 206, 1120 ± 571 and 868 ± 441, respectively. Thus, Herd 2 had the highest mean level of passively acquired anti-BCV antibodies, but also the largest range of antibody titers (Fig. 5a).

Anti-BCV serum antibody levels were also measured over time in 60 randomly selected study calves from each herd at initial vaccination, preconditioning, and weaning. The calves evaluated from Herd 3 had the highest mean antibody abundance (489 ± 386) at initial vaccination, which was significantly greater than Herd 1 (285 ± 117) but did not differ from Herd 2 (400 ± 222). At preconditioning, Herd 2 had the highest mean antibody abundance (191 ± 89) that was significantly greater than Herds 1 (148 ± 109) and 3 (138 ± 56). Herd 2 continued to have the highest mean antibody abundance at weaning (163 ± 73), which was significantly greater than Herds 1 (103 ± 78) and 3 (113 ± 52; Fig. 5a).

To determine whether anti-BCV antibody levels were predictive for which individuals in Herd 2 would develop signs of respiratory disease, antibody levels from Figs. 4 and 5a were re-plotted to compare mean antibody abundances between: a) those mass treated for BRD with a rectal temperature ≥ 39.4 °C, b) those mass treated for BRD with a rectal temperature < 39.4 °C, and c) those from the same herd that remained untreated for BRD throughout the study. No statistically significant difference in mean antibody abundance was observed within sample acquisition times for these three groups (*P* = 0.48; Fig. 5b). In summary, BCV antibody abundance was not associated with pre-weaning BRD incidence within or between herds.

### Subclinical shedding of BCV was detected in all three herds

To evaluate pathogen exposure, subclinical respiratory shedding of BCV as well as BHV-1, BRSV and BVDV was measured in the 60 randomly selected calves from each herd evaluated for anti-BCV antibody levels at initial vaccination, preconditioning and weaning. Bovine viral diarrhea virus, BHV-1 and BRSV were not detected in any calf at any sample acquisition time. Bovine coronavirus was detected in nasal secretions from two (3%), 10 (17%), and two (3%) of the 60 calves of Herds 1, 2, and 3, respectively, at preconditioning; and one calf from Herd 2 at weaning (Table 3). Thus, BCV was detected in all three herds and shedding was highest at preconditioning processing, with an overall prevalence across herds of 8% (14/180).

**Table 3:** Subclinical shedding of BCV

Sample Acquisition Time	Number of BCV shedding calves/total evaluated (% positive)			
	Herd			Total
	1	2	3	
Initial vaccination	0/60 (0)	0/60 (0)	0/60 (0)	0/180 (0)
Preconditioning	2/60 (3.3)	10/60 (16.7)	2/60 (3.3)	14/180 (7.7)
Weaning	0/60 (0)	1/60 (1.7)	0/60 (0)	1/180 (0.6)
Total	2/180 (1.1)	11/180 (6.1)	2/180 (1.1)	15/540 (2.8)

Of the 60 randomly selected calves from Herd 2, 45 were involved in one of the mass treatment events for BRD and 15 were not. Seven of 45 (15.6%) calves that were previously treated for BRD were shedding BCV at preconditioning. Similarly, three of 15 (20%) that were not previously treated for BRD were shedding BCV at preconditioning. The one calf shedding BCV at weaning was treated previously for BRD.

### Anti-BCV antibody abundance did not associate with respiratory shedding of BCV

**Fig. 6:**
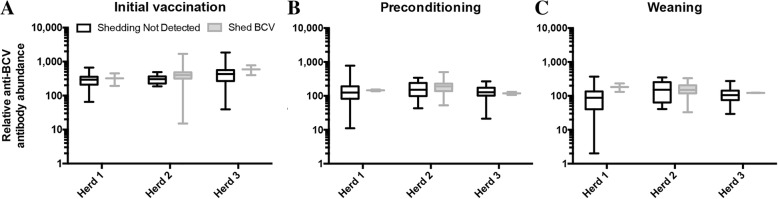
Relationship between anti-BCV antibody abundance and respiratory virus shedding. Anti-BCV antibody abundances from Fig. 5a were re-plotted to compare anti-BCV antibody abundance between calves that shed BCV at some point during the pre-weaning period (n = 2, 48, and 2 for Herds 1, 2, and 3, respectively) to those animals where BCV shedding was never detected (*n* = 58, 12, and 58 for Herds 1, 2, and 3, respectively). **a **initial vaccination, **b** preconditioning, **c** weaning. Differences in mean antibody abundances were evaluated by testing location, BCV shedding status, and their interaction, and no significant difference was observed within herds at any production stage (*P* > 0.05)

To determine how serum anti-BCV antibody levels related to respiratory BCV shedding, anti-BCV antibody levels from Fig. 5b were re-plotted to compare antibody levels between those found to be shedding BCV during the pre-weaning period and those that were not. Within herds, claves found to be shedding BCV at mass treatment, preconditioning, or weaning did not have significantly different relative anti-BCV antibody abundances compared to the rest of the herd when measured at initial vaccination (prior to when they shed BCV) or at preconditioning (Fig. 6). At weaning, there was a trend for Herd 1, where the calves that were shedding BCV at preconditioning had among the highest anti-BCV antibody abundances; however, the sample size was extremely small (*n* = 2). Therefore, there was no statistically significant difference in the mean antibody abundance for those found to be shedding BCV and those that were not shedding BCV at each production stage.

### Sequence analysis of the BCV spike gene

To compare the BCV strain(s) circulating in the three herds, a fragment of the BCV spike gene spanning the S1 hypervariable domain and antigenic domain II was analyzed. Fifteen positive nasal swabs were analyzed: six were from Herd 2 at the time of mass treatment (two from 8/5/16 and four from 8/12/16), eight from preconditioning (two from Herd 1, four from Herd 2 and two from Herd 3), and one from weaning (Herd 2). A total of four SNPs (compared to the consensus) were identified in three samples, each leading to a change in the predicted amino acid at that position (Table 4). Of these, the first three amino acid changes are located within the hypervariable domain and the last two are located in antigenic domain II, which partially overlaps with the hypervariable region [30].

**Table 4:** Sequence analysis of the BCV spike gene. Sequence analysis of a 1102 nucleotide fragment of the BCV spike gene spanning the S1 hypervariable region and antigenic domain II

	Position in Mebus spike peptide (U00735.2)^a^															
	458	465	470	476	484	492	509	510	525	531	535	543	546	571	578	628
Mebus	F	V	H	A	S	D	N	S	H	N	T	S	P	Y	T	L
1701_PC_1^b^	S	A	D	A	T	G	T	T	Y	D	T	A	S	H	S	L
2018_PC_1	S	A	D	A	T	G	T	T	Y	D	T	A	S	H	S	L
2507_PC_2	S	A	D	**V**	T	G	T	T	Y	D	T	A	S	H	S	L
2369_PC_2	S	A	D	A	T	G	T	T	Y	D	T	A	S	H	S	L
2785_PC_2	S	A	D	A	T	G	T	T	Y	D	T	A	S	H	S	L
2830_PC_2	S	A	D	A	T	G	T	T	Y	D	T	A	S	H	S	L
1399_PC_3	**F** ^**c**^	A	D	A	T	G	T	T	Y	D	T	A	S	H	S	**F**
4150_PC_3	S	A	D	A	T	G	T	T	Y	D	**I**	A	S	H	S	L
2402_MT_2	S	A	D	A	T	G	T	T	Y	D	T	A	S	H	S	L
2507_MT_2	S	A	D	A	T	G	T	T	Y	D	T	A	S	H	S	L
2812_MT_2	S	A	D	A	T	G	T	T	Y	D	T	A	S	H	S	L
2815_MT_2	S	A	D	A	T	G	T	T	Y	D	T	A	S	H	S	L
2826_MT_2	S	A	D	A	T	G	T	T	Y	D	T	A	S	H	S	L
2830_MT_2	S	A	D	A	T	G	T	T	Y	D	T	A	S	H	S	L
2721_W_2	S	A	D	A	T	G	T	T	Y	D	T	A	S	H	S	L

^a^Inferred polypeptide sequences were aligned with the reference Mebus genome (Genbank accession U00735.2) corresponding to amino acids 396 to 762, and only those amino acid positions differing from the reference Mebus strain are pictured

^b^Strains from study calves are listed as: animal ID_sample acquisition time_herd number. PC = preconditioning, MT = mass treatment, W = weaning

^c^Bold letters are the four amino acid changes inferred by the single nucleotide polymorphisms identified by Sanger sequencing

BCV strains from two of the animals from Herd 2 were sequenced at two different sample acquisition times. The BCV strain(s) infecting animal 2507 differed by a single nucleotide between the two time points. In contrast, the BCV strain(s) infecting animal 2830 was identical at this location at both collection points. Furthermore, the strain present in Herd 2 was identical to the strain in Herd 1 when comparing this region, and only one or two SNPs were present in the strains circulating in Herd 3. Thus, the strains present in the three herds were very similar when analyzing the hypervariable region of the spike gene, and some animals were persistently infected or re-infected with the same or similar strain during the course of the study.

## Discussion

This study represents the first prospective longitudinal study of the relationship between anti-BCV antibody levels and BCV infections and respiratory disease in nursing beef calves. Two outbreaks of respiratory disease in one research herd resulted in two-thirds of the calves being mass treated for BRD. BCV shedding was found to occur in conjunction with the pre-weaning BRD outbreak. However, when levels of passively or actively acquired immunity to BCV were measured, they were not found to associate with respiratory disease incidence between the three study herds, nor with the development of BRD in individual calves within that herd. Rather, BCV infections were likely common, with many subclinical and some persistent or recurrent infections detected.

The results from this study agree with the results from previous studies in young dairy calves [16, 17]. In these studies, dairy calves shed virus, sometimes repeatedly, in spite of relatively high anti-BCV antibody titers. Furthermore, they observed no statistically significant correlation between maternal BCV specific IgG serum antibody titers and clinical disease or infection by BCV in that population [16]. Similarly, in the study reported here, we found that BCV specific serum IgG did not correlate with respiratory disease; and 8/45 calves (18%) from Herd 2 that were involved in mass treatment were found to be shedding BCV at two or more sample acquisition times. This result supports the idea that persistent or recurrent shedding episodes can occur in the same animal with or without signs of disease.

BCV infection (as measured by virus shedding) was similarly not found to be associated with anti-BCV antibody levels; however, this measurement was hampered by the infrequency of sample collection in our study design (Fig. 1). For example, nearly all of the animals in the Herd 2 mass treatment groups shed BCV between our routine sample collection times. While we did not see differences in anti-BCV antibody abundances in Herd 2 between those calves mass treated for BRD and those from the same herd that remained untreated, it is possible, and even likely, that many untreated calves also shed BCV between sample acquisition dates. Lack of BCV detection in nasal swabs at routine collection times may lead to “false negatives” for BCV infection that mask any influence antibodies may have on virus shedding. Furthermore, the small number of BCV positive individuals found in Herds 1 and 3 (two calves from each herd were shedding BCV at preconditioning processing) hinder our ability to detect with any confidence differences that may exist. More intensive sampling would be required to better determine the association between anti-BCV antibody titers and BCV infection. Other hypothetical reasons why serum anti-BCV IgG abundance has not been found to associate with BCV infection or treatment for disease has been discussed previously [15, 16]. Among these is the lack of knowledge related to the immune correlates of protection for respiratory BCV infections. How cell mediated immunity and other antibody isotypes (such as IgA) contribute to protection from infection and disease are areas that require additional research.

Given that BCV infection is common, it remains unknown why some animals display signs of respiratory disease while others remain sub-clinically infected. To determine whether there were differences in the BCV strains circulating in the three herds, a 1102 nucleotide fragment of spike gene was analyzed. The spike gene encodes the surface glycoprotein that is responsible for attachment to the host cell and is a major neutralizing antigen targeted by the host immune system [34, 35]. The region of the spike gene spanning the hypervariable region and antigenic domain II was selected because it is variable between coronavirus strains and isolates, and variations in this region have been associated with altered antigenicity and/or pathogenicity in other species [6]. No polymorphisms were found in this region between Herd 1 and Herd 2 or between the isolates from subclinical shedding episodes compared to the isolates circulating at the time of the BRD outbreak. Furthermore, only one or two SNPs were found between the isolates circulating in Herd 3 compared to the isolates in Herds 1 and 2. In contrast, up to 35 SNPs and 11 amino acid differences were detected in this same region when these isolates were compared to 14 isolates collected between 2014 and 2017 from USMARC and the University of Nebraska-Lincoln Veterinary Diagnostic Center (unpublished data). Thus, differences in the BCV strains circulating in the three herds are unlikely to account for the major differences in treatment rates observed in this study.

One side effect of respiratory viral infection is the increased risk for bacterial superinfection [4]. While BCV may occasionally produce a clinical syndrome consistent with BRD in the absence of bacterial infection, its involvement, like other respiratory viruses, is generally considered to be a precursor to a bacterial infection, which exacerbates disease [2, 4, 36]. Thus, the presence of co/secondary bacterial infections may help explain the differences in disease severity in BCV-infected calves observed in this study. Using qPCR to look at common bacterial pathogens associated with respiratory disease in cattle, we observed *H. somni* in high frequency and abundance in the upper respiratory tract of sick cattle but not clinically normal cattle from the same herd collected 2–3 weeks after the outbreak. Furthermore, *H. somni* was not detected in these calves at initial vaccination, prior to the disease outbreak. Thus, we hypothesize that a potential secondary bacterial infection with *H. somni* may explain why the cattle in Herd 2 displayed more signs of respiratory disease and subsequently required treatment. Of note, however, within treatment groups there was no difference in the relative abundance of *H. somni* in the nasal cavity of animals when categorized by rectal temperature taken at the time of sample collection and treatment. This suggests that infection was widespread in the herd, though only 15–30% of the animals were displaying clinical signs of disease at that particular time. Frequency differences of various strains of these bacteria that have different propensities to cause disease may have been present between these populations, but were not differentiated by the assays used. It is also possible that additional pathogens that were not measured in this study could have contributed to the disease outbreak. Thus, unbiased metagenomic approaches are currently underway to determine whether any additional viral or bacterial pathogens were associated with the disease outbreak. How the URT microbiome may have influenced respiratory health is also being examined in these populations.

## Conclusions

Co-detection of BCV and *H. somni* at the time of the disease outbreak suggests that these two pathogens contributed to disease pathogenesis. Characterizing the factors that may have contributed to the outbreak described here can help veterinarians, researchers, and producers better understand the risk factors associated with pre-weaning BRD. This information can be used to guide prevention and control strategies to decrease the incidence of BRD and associated adverse animal health issues and production losses.

## Additional files

Additional file 1: Bovine respiratory disease cases. (XLS 29 kb)

Additional file 2: Comparison of antibody detection assays for bovine coronavirus. An ELISA for the detection of total reactive antibodies against BCV was compared with the VNT using 60 plasma or serum samples collected between birth and weaning from 12 calves to determine whether the commercially available ELISA could be used as a reliable substitute for the VNT to measure BCV immunity in this population. The effect of altering the strain of the test virus used in the VNT was also evaluated. A Pearson’s correlation coefficient (ρ) was derived between the results obtained by VNT and ELISA and between the VNT using Mebus and BRCV_2014 challenge strains. (TIF 117 kb)

## Abbreviations

BCV: Bovine coronavirus
BHV-1: Bovine herpesvirus 1
BRD: Bovine respiratory disease
BRSV: Bovine respiratory syncytial virus
BVDV: Bovine viral diarrhea virus
Ct: Cycle threshold
ELISA: Enzyme-linked immunosorbent assay
MLV: Modified live virus
qPCR: Real-time polymerase chain reaction
RT-qPCR: Reverse transcription real-time polymerase chain reaction
SD: Standard deviation
URT: Upper respiratory tract
USMARC: U.S. Meat Animal Research Center
VNT: Virus neutralization test
