# Supplementary figures and images for: Longitudinal study of humoral immunity to bovine coronavirus, virus shedding, and treatment for bovine respiratory disease in pre-weaned beef calves
Source: BMC Vet Res. 2019 May 22;15:161. doi: 10.1186/s12917-019-1887-8 (PMC6532244; doi:10.1186/s12917-019-1887-8)

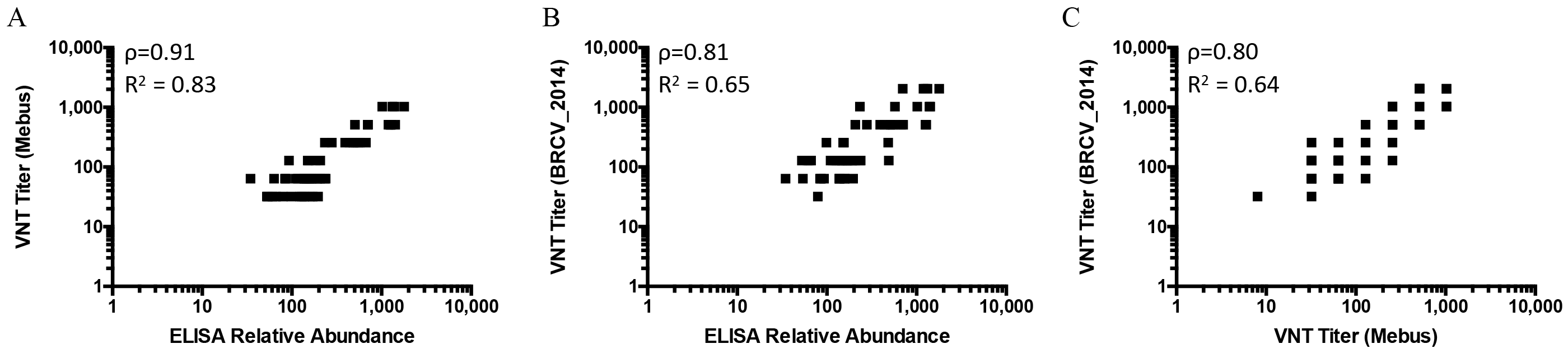

Supplement: Supplementary file 2 — Comparison of antibody detection assays for bovine coronavirus. An ELISA for the detection of total reactive antibodies against BCV was compared with the VNT using 60 plasma or serum samples collected between birth and weaning from 12 calves to determine whether the commercially available ELISA could be used as a reliable substitute for the VNT to measure BCV immunity in this population. The effect of altering the strain of the test virus used in the VNT was also evaluated. A Pearson’s correlation coefficient (ρ) was derived between the results obtained by VNT and ELISA and between the VNT using Mebus and BRCV_2014 challenge strains. (TIF 117 kb) [file 12917_2019_1887_MOESM2_ESM.tif]
